# Chemical Composition and Evaluation of the *α*-Glucosidase Inhibitory and Cytotoxic Properties of Marine Algae *Ulva intestinalis, Halimeda macroloba*, and *Sargassum ilicifolium*

**DOI:** 10.1155/2020/2753945

**Published:** 2020-11-22

**Authors:** Muhammad Farhan Nazarudin, Azizul Isha, Siti Nurulhuda Mastuki, Nooraini Mohd. Ain, Natrah Fatin Mohd Ikhsan, Atifa Zainal Abidin, Mohammed Aliyu-Paiko

**Affiliations:** ^1^Laboratory of Aquatic Animal Health and Therapeutics, Institute of Bioscience, Universiti Putra Malaysia, Serdang 43400, Selangor, Malaysia; ^2^Laboratory of Natural Medicines and Products Research, Institute of Bioscience, Universiti Putra Malaysia, Serdang 43400, Selangor, Malaysia; ^3^Laboratory of UPM-MAKNA Cancer Research, Institute of Bioscience, Universiti Putra Malaysia, Serdang 43400, Selangor, Malaysia; ^4^Department of Aquaculture, Faculty of Agriculture, Universiti Putra Malaysia (UPM), Serdang 43400, Selangor, Malaysia; ^5^Biochemistry Department, Ibrahim Badamasi Babangida University (IBBU), Lapai, Nigeria

## Abstract

Seaweed has tremendous potentials as an alternative source of high-quality food products that have attracted research in recent times, due to their abundance and diversity. In the present study, three selected seaweed species commonly found in the Malaysian Peninsular, *Ulva intestinalis, Halimeda macroloba*, and *Sargassum ilicifolium,* were subjected to preliminary chemical screening and evaluated for *α*-glucosidase inhibitory and cytotoxic activities against five cancer cell lines. Chemical composition of *U. intestinalis, H. macroloba,* and *S. ilicifolium* methanolic extracts was evaluated by Gas Chromatography-Mass Spectrometry (GC-MS) analysis. Our results revealed the highest total carotenoids (162.00 *μ*g g^−1^ DW), chlorophyll *a* (313.09 ± 2.53 *μ*g g^−1^ DW), and chlorophyll *b* (292.52 ± 8.84 *μ*g g^−1^ DW) concentrations in *U. intestinalis*. In the *α*-glucosidase inhibitory activity, *S. ilicifolium* demonstrated the lowest efficacy with an IC_50_ value of 38.491 ppm compared to other species of seaweed. *H. macroloba* extract, on the other hand, was found to be the most cytotoxic toward MCF-7 and HT 29 cells with IC_50_ of 37.25 ± 0.58 and 21.32 ± 0.25 *μ*g/mL, respectively, compared to other cell lines evaluated. Furthermore, *H. macroloba* extract was also found to be less toxic to normal cell (3T3) with IC_50_ of 48.80 ± 0.11 *μ*g/mL. *U. intestinalis* extract exhibited the highest cytotoxicity toward Hep G2 cells with IC_50_ of 23.21 ± 0.11 *μ*g/mL, whereas *S. ilicifolium* was less toxic to MDA- MB231 cell with IC_50_ of 25.23 ± 0.11 *μ*g/mL. Subsequently, the GC-MS analysis of the methanolic extracts of these seaweed samples led to the identification of 27 metabolites in *U. intestinalis*, 22 metabolites in *H. macroloba*, and 24 metabolites in *S. ilicifolium*. Taken together, the results of this present study indicated that all the seaweed species evaluated are good seaweed candidates that exhibit potential for cultivation as functional food sources for human consumption and need to be promoted.

## 1. Introduction

Marine seaweed is a diverse group of marine resources that are commonly found in the maritime regions of the world. Seaweed also epitomizes a lavish cradle of natural products, containing highly treasured chemicals that have not been properly investigated for their valuable compositions. Globally, trade in macroalgae (seaweed) is a multibillion-dollar industry [1] with world production of seaweed increasing between 1970 and 2010 from <2 million to 19 million tonnes fresh weight [2]. Recent uses of seaweed include human food, fertilizers, phycocolloids, and cosmetic ingredients for nutraceuticals and pharmaceuticals [3–6]. Nonetheless, seaweed is still considered an underutilized resource worldwide [7].

In research and development, most researchers working on marine seaweed are those within the medical or pharmacology disciplines, where their attention is particularly focused on the medical significance and application of extracts from seaweed based on pharmaceutical potentials [8]. This is because marine natural products have long been used to prevent and treat many diseases, thus making them good candidates for the development of anticancer drugs [9] and the management of other maladies like diabetes. At the moment, cancer and diabetes mellitus, especially Type 2 diabetes mellitus (T2DM) are among the group of diseases that are considered to be of public health concern around the world. Moreover, the prevalence of these diseases has increased steadily in recent years [10]. In Malaysia, for instance, data from the National Health and Morbidity Survey (NHMS) showed the prevalence of T2DM as significant, measured at approximately 14.9% in 2006. This alarming public health concern continues to increase, as in 2011 and 2015, 15.2% and 17.7% cases were recorded, respectively. The prevalence of T2DM in Malaysia is projected to reach about 21.6% in 2020. As the use of natural anticancer and antidiabetic medication in effective chemopreventive therapy is fast becoming one of the important approaches of cancer prevention and for management of T2DM, the evaluation for the active components responsible for the biological activities against these ailments needs to be properly investigated.

Seaweed is acknowledged for its excellent content of fucoidan, phenolics, fatty acids, polysaccharides, vitamins, sterols (such as fucosterol in brown seaweed), tocopherols, terpenoids, and phycocyanins, among others [11–13]. In addition to fatty acids, the unsaponifiable fractions of seaweed contain major photosynthetic pigments. The major photosynthetic pigments studied in seaweed, including chlorophylls (*a, b, c*), carotenoids (such as beta-carotene, lutein, and violaxanthin in red and green seaweed, fucoxanthin in brown seaweed), and phycobilins (phycoerythrin and phycocyanin) have been acknowledged to exhibit various benefits for human prosperity. This is especially true in terms of their uses as food, alternative treatments, and medicine including antioxidants, anticancer, anti-inflammatory, antidiabetic, antiobesity, antiangiogenic, and neuroprotective activities [14–16].

It has been suggested, therefore, that further work needs to be performed to identify the metabolites components of seaweed that are useful for industrial and pharmaceutical applications. A holistic approach is proposed to further authenticate the health benefits of seaweed, in order to unravel the new potentials of edible species through biochemical profiling. Consequently, the objectives of the present study were to evaluate the chemical compositions and investigate the *α*-glucosidase inhibitory and cytotoxic activities of extracts of 3 selected species of seaweed available in Peninsular Malaysia against five cancer cell lines including MCF-7 cells, MDA-MB-231 cells, HT-29 cells, Hep G2 cells, and 3T3 cells using a colorimetric (MTT) assay. The three selected species of seaweed investigated include *U. intestinalis, H. macroloba*, and *S. ilicifolium*. The overall aim of this study was to explore the potentials of these abundant species of seaweed for utilization as a functional food for human consumption, in order to promote the commercial cultivation of the species.

## 2. Materials and Methods

### 2.1. Sample Collection and Preparation

**Figure 1:**
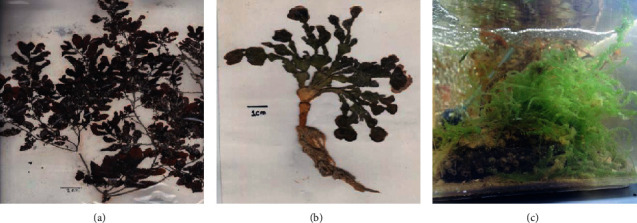
Seaweed samples. (a) *S. ilicifolium*. (b) *H. macroloba*. (c) *U. intestinalis*.

All seaweed samples were collected between September 2015 and September 2016 during low tides (<0.09 meters) from three different locations in Malaysia as shown in Table 1 and Figure 1. All samples were collected based on the locations where they were most abundant. Collected seaweed samples were cleaned of extraneous materials like epiphytes, sand particles, pebbles, and shells using a soft bristle brush and washed thoroughly with seawater. The samples were divided into two portions. The first portion was taken into the herbarium for taxonomic identification. The taxonomic identification was done by cross-referencing with taxonomic books, monographs, and reference herbaria.

The second portion of the clean samples was then placed in transparent polyethylene bags (inside chilled plastic containers) and transported to the research facilities of the Laboratory of Marine Biotechnology, Institute of Bioscience (IBS), Universiti Putra Malaysia (UPM) Serdang, Malaysia. In the laboratory, the samples were further washed thoroughly with tap water, followed by distilled water, and frozen overnight at −80°C in a deep freezer (Thermo Scientific, USA). Frozen seaweed samples were subsequently lyophilized in a freeze dryer (Labconco FreeZone, USA) until a constant weight of biomass was attained. All the dried seaweed samples were then pulverized into a fine powder with a laboratory-scale blender and sieved using a 200 *μ*m sized sieve.

Fine sample powders were collected and mixed with methanol (1 : 20 w/v) to make a solution. This resulting solution was extracted by sonication for 30 min using an ultrasonic water bath (Power Sonic 505, Korea) at ambient temperature. The resulting solvent extract was then filtered through Whatman No. 1 filter paper, whereas the dried residue was mixed and reextracted with another fresh methanol for the second round of extraction. The extraction process was repeated in the same manner for the third round of extraction. The filtered extracts were pooled and evaporated to dryness using a rotary evaporator, maintained at 40°C. The concentrated extracts were stored in amber bottles in a freezer for future utilization.

### 2.2. Determination of Total Carotenoids and Chlorophyll Contents

Lipid extraction was performed on the finely powdered seaweed samples according to slight modifications to the method adopted by Sun et al. 2019 [17]. Briefly, approximately 0.5 g of freeze-dried seaweed powder was homogenised (Branson CPX2800H Ultrasonic Cleaner, USA) with 20 mL of chloroform: methanol (2 : 1 v/v) for 5 min. The homogenate was filtered on Whatman No.1 filter paper. The filtrate was mixed with 10 mL aqueous solution of 9% sodium chloride and subsequently centrifuged (Eppendorf 5810R, Germany) at 2000 rpm for 8 min, which differentiated into two layers: an upper aqueous layer and a lower organic layer. The lower (organic) layer containing chloroform was collected from the separation apparatus. The residual biomass recovered from the filtration step was again subjected to repeated rounds of solvent extraction of lipids (three times), in order to recover as many lipids as possible. The collected chloroform fractions from the multiple extraction procedure were pooled together per sample and concentrated, using a rotary evaporator (N-1001S-WD, with EYELA Oil Bath OSB-2000, Japan). A concentrated lipid extract was then left to dry in a vacuum oven (Memmert, USA) maintained at 40°C, until a constant weight was attained. Dry lipid samples were kept in amber coloured sample bottles fitted with screw caps and stored in desiccators, at ambient temperature (25°C) until used for further analysis immediately, or at −20°C for longer storage. Total carotenoids and chlorophyll *a* and *b* contents of dried seaweed lipid extracts were determined in accordance with slight modifications to the method adopted by Lichtenthaler and Buschmann in 2001 [18]. According to this method, dilution in methanol was carried out of each seaweed lipophilic extract and the absorbance of total carotenoids; chlorophyll *a* and chlorophyll *b* were measured at 470, 665.2, and 652.4 nm, respectively, using a UV-spectrophotometer (Shimadzu UV 1601, Japan). Calculations of the total carotenoids and chlorophyll *a* and chlorophyll *b* contents in each seaweed lipid sample were performed using the Lichtenthaler equations as follows (with values expressed as *μ*g g^−1^ dry weight [*μ*g g^−1^ DW]):

(1) $$\begin{array}{c} C_{\left(x+c\right)}\left(\mu gg^{-1}\right)=\frac{\left(1000A_{470}-1.63C_{a}-104.96C_{b}\right)}{221},\\ C_{a}\left(\mu gg^{-1}\right)=16.72A_{665.2}-9.16A_{652.4},\\ C_{b}\left(\mu gg^{-1}\right)=34.09A_{652.4}-15.28A_{665.2}, \end{array}$$

where *C*_(*X*+*C*)_ is the total carotenoids, *C*_*a*_ is chlorophyll a, *C*_*b*_ is chlorophyll b, *A*_470_ is the absorbance at 470 nm, *A*_665.2_ is the absorbance at 665.2 nm, and *A*_652.4_ is the absorbance at 652.4 nm.

### 2.3. *α*-Glucosidase Inhibition Assay

The assay for *a*-glucosidase inhibition activity was performed as described by Lee et al. (2014) [19]. The substrate used was *ρ*-Nitrophenyl-*ρ*-D-glucopyranoside (PNPG), prepared by dissolving in 50 mM phosphate buffer (pH 6.5) that is comparable to the conditions of the human intestinal fluid. The seaweed extracts were prepared at a concentration of 5000 ppm and 6 serial dilutions were performed. The extracts were mixed in the 96-well microplate and incubated at room temperature for 5 min. Then, 75 *μ*L of PNPG was added to each well containing the sample, blank substrate, and negative or positive controls whereas the remaining wells were loaded with 75 *μ*L of 30 mM phosphate buffer. These mixtures in the wells were incubated for 15 min at ambient temperature. The reaction mixtures were stopped by using the stopping agent, 50 *μ*L of 2 M glycine (pH 10) for the sample, blank substrate, and negative control. To the remaining wells was added 50 *μ*L of deionized water. Subsequently, the absorbance readings for all the wells were measured using a spectrophotometer (Tecan Infinite F200) at a wavelength of 405 nm. The *a*-glucosidase inhibition activity of the test sample was expressed as a percentage (%) of inhibition and calculated using the following:

(2) $$\begin{array}{c} \%\text{ inhibition of sample}=\left[\frac{\left(\text{Negative control }–\text{ Blank negative control }\right)–\left(\text{Sample }–\text{ Blank Sample}\right)}{\left(\text{Negative control }–\text{ Blank negative control}\right)}\right]x100\%. \end{array}$$

### 2.4. Cell Lines Used, Source, and Culture Conditions

MCF-7 cells (human breast adenocarcinoma cell line, estrogen receptor positive), MDA-MB-231 cells (human breast adenocarcinoma cell line, estrogen receptor negative), HT-29 cells (human colorectal adenocarcinoma cell line), Hep G2 cells (human liver hepatocellular carcinoma cell line), and 3T3 cells (mouse embryonic fibroblast cells) were kindly provided by the Laboratory of UPM - MAKNA Cancer Research, Institute of Bioscience, Universiti Putra Malaysia. Cells were maintained in RPMI medium supplemented with 10% fetal calf serum (FCS), 100 unit/ml penicillin, and 0.1 mg/ml streptomycin.

### 2.5. MTT Assay Conditions

The methanolic extracts of the three species of seaweed evaluated, *U. intestinalis, H. macroloba*, and *S. ilicifolium,* were tested further for their cytotoxicity levels. The extracts were tested on MCF-7 cells (human breast adenocarcinoma cell line, estrogen receptor positive), MDA-MB-231 cells (human breast adenocarcinoma cell line, estrogen receptor negative), HT-29 cells (human colorectal adenocarcinoma cell line), Hep G2 cells (human liver hepatocellular carcinoma cell line), and 3T3 cells (mouse embryonic fibroblast cells). All cells were maintained in RPMI medium supplemented with 10% fetal calf serum (FCS), 100 unit/ml penicillin, and 0.1 mg/ml streptomycin. Cytotoxicity of the extracts was determined via MTT assay according to the protocol utilized by Mohan et al. 2008 [20], using 3-[4, 5-dimethylthiazol-2-yl]-2,5-diphenyltetrazolium bromide. Initially, cell culture at a concentration of 1 × 10^5^ cells/mL was prepared and plated (100 *μ*L/well) into 96-well plates. The cell was incubated for 24 h at 37°C, 5% CO_2_. Diluted ranges of extract samples (1.56, 3.125, 6.25, 12.5, 25, 50, and 100 *μ*g/mL) were dissolved in dimethyl sulfoxide (DMSO), and the final concentration of DMSO was 0.1% (v/v). Thereafter, various concentrations of the extract samples were plated out in triplicate. Each plate included untreated cell controls and blank cell-free control. 5-Fluorouracil, a drug with antineoplastic activity, was used as a positive control in this study. After 68 h of incubation (to allow for the cancer cells to grow properly or to be inhibited based on extract cytotoxicity), 20 *μ*L of MTT (5 mg/mL) was added to each well to form formazan crystals, and the plates were incubated for further 4 h and the media was removed. DMSO was later added to each well to solubilize the formazan crystals. The absorbance was read at a wavelength of 570 nm using a microplate reader (Tecan Sunrise Basic, Groedig, Austria). The percentage of cellular viability was calculated with the appropriate controls taken into account. The concentration which inhibited 50% of the cellular growth (IC_50_ value) was determined. The inhibitory rate of cell proliferation was calculated by the following formula: growth inhibition = (OD control−OD treated)/OD control×100. The cytotoxicity of the sample on cancer cells was expressed as IC_50_ values (the sample concentration reducing the absorbance of treated cells by 50% with respect to untreated cells). All cultures were tested for mycoplasma contamination and were found to be negative.

### 2.6. Gas Chromatography-Mass Spectrometry Analysis

Gas Chromatography-Mass Spectrometry (GC-MS) analysis was employed to characterize and quantify bioactive substances in the seaweed extracts. Methanolic extracts were characterized quantitatively via GC-MS, according to slight modifications to the method adopted by Han et al. 2009 [21], using a Shimadzu QP2010 Plus GC-MS system. In the experimental procedure, 0.5 *μ*L of the sample was separated on a Zebron ZB5-ms 30 m × 0.25 mm ID *x* 0.25 *μ*m film thickness) column. The splitless injection was performed using a purge time of 1 min. Helium represented the carrier gas at a flow rate of 1 mL/min. The column temperature was maintained at 50°C for 3 min, then programmed at 250°C for 10 min, and maintained at 250°C for 30 min. The inlet and the detector temperatures were set at 250°C, and the solvent cut time was set at 4.50 min. The identification of peaks was based on a computer-based program matching the mass spectra with those in the library for the National Institute of Standards and Technology (NlST3208 and NIST 08s). This was done by comparing retention time data with that obtained for authentic laboratory standards. Individually detected peak areas were quantified and expressed as a percentage of total components detected.

### 2.7. Statistical Analysis

All data are expressed as mean ± standard deviation of three replicates for total carotenoids, chlorophylls *a* and *b,* and *α*-glucosidase inhibition assay. For MTT assay, data are presented as mean values (and their standard errors) of six replicate determinations in either tables or figures, showing error bars to represent deviations. All data for total carotenoids, chlorophylls *a* and *b,* and *α*-glucosidase inhibition assay and MTT assay were analysed by one-way analysis of variance (ANOVA), and the mean of replicate measurements was taken. All values of *P* < 0.05 were considered significant, at a 95% confidence level.

## 3. Results

The major photosynthetic pigments, total carotenoids content, and chlorophyll *a* and *b* in seaweed samples studied are shown in Table 2 and expressed as *μ*gg^−1^ dry weight (DW). From the results, the total concentration of carotenoids was comparable and significantly (*P* < 0.05) the highest in *U. intestinalis* (162.00 ± 0.84 *μ*gg^−1^ DW), followed by *H. macroloba* (117.36 ± 1.30 *μ*gg^−1^ DW). Total carotenoid content was detected as significantly the lowest in *S. ilicifolium* (45.28 ± 1.77 *μ*gg^−1^ DW) among the seaweed species studied. Present data show that chlorophyll *b* was the richest in green algae, *U. intestinalis* (292.52 ± 8.84 *μ*gg^−1^ DW), and the lowest chlorophyll *b* data was measured in brown algae, *S. ilicifolium* (111.29 ± 2.28 *μ*gg^−1^ DW).

Table 3 shows the inhibition activities of the various seaweed species on the *a*-glucosidase enzyme. Methanolic extract of brown seaweed *S. ilicifolium* showed significantly (*P* < 0.05) lower IC_50_ value of 38.49 ppm, followed by *H. macroloba* with an IC_50_ value of 71.77 ppm, while *U. intestinalis* recorded IC_50_ value above 200 ppm. The control (quercetin) showed significant effects of *a*-glucosidase activity with the lowest IC_50_ value of 16.53 ppm.

**Figure 2:**
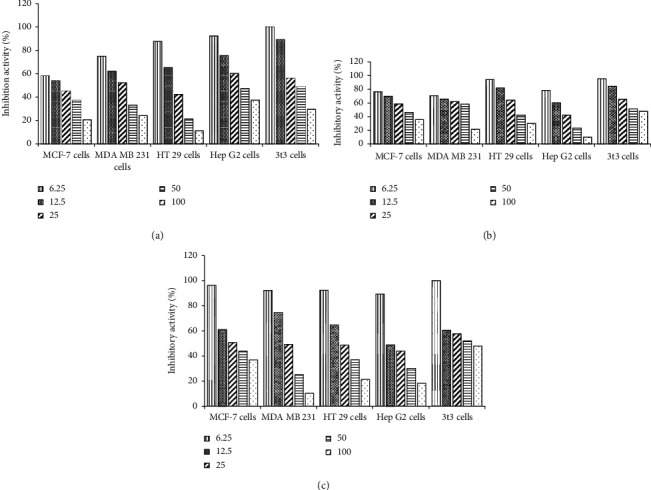
Cytotoxic activities of methanolic extract of (a) *H. macroloba*, (b) *U. intestinalis*, and (c) *S. ilicifolium* at different concentrations (6.25–100 *μ*g/ml against five different cancer cell lines in an MTT assay.

The determination of cytotoxicity was carried out using a dose-response curve obtained by nonlinear regression analysis. The cell viability was determined by comparison to the survival of cells in the untreated (negative control) cultures, which was normalised to 100%. Our data indicated the potential cytotoxic activity of the methanolic extract for both green seaweed species *U. intestinalis* and *H. macroloba* and brown seaweed *S. ilicifolium* on five different cell lines, MCF 7, MDA- MB231, Ht-29, Hep G2, and 3T3 cells, as shown by the IC_50_ results in Table 4 and Figure 1. The results show a clear decrease in cell viability and cell growth inhibition in a dose-dependent manner. As shown in Table 4 and Figure 2, *H. macroloba* extract was found to be most cytotoxic towards MCF-7 and HT 29 cells with IC_50_ 37.25 ± 0.58 and 21.32 ± 0.25 *μ*g/mL, respectively, compared to other cell lines. Besides, *H. macroloba* extract was also the least toxic to normal cell (3T3) with IC_50_ of 48.80 ± 0.11 *μ*g/mL, compared to the other extracts evaluated. *U. intestinalis* extract exhibited the most cytotoxicity towards Hep G2 cells with IC_50_ 23.21 ± 0.11 *μ*g/mL, while *S. ilicifolium* was less toxic towards MDA- MB231 cell with IC_50_ 25.23 ± 0.11 *μ*g/mL.

GC-MS is a combined method that is applied to identify different chemical contents within a sample. It functions on the separation of the individual compounds by gas chromatography according to their retention time and the separated compounds are further analysed at a molecular level by mass spectrometry. The GC-MS profiling of methanolic extracts resulted in the identification of 27 metabolites in *U. intestinalis*, 22 metabolites in *H. macroloba,* and 24 metabolites in *S. ilicifolium*. These metabolites belong to different chemical classes, and most of them are reported to exhibit important biological activities. The metabolites also included various aliphatic acids and aromatic compounds identified in the different samples. The identified compounds with their retention time (RT), peak area (%), and molecular formula are presented in Tables 5–7. Out of 27 peaks observed in the chromatogram of *U. intestinalis* methanolic extract, five compounds were identified as major peaks. The major compounds included 1,2-benzenedicarboxylic acid, mono(2-ethylhexyl) ester (7.61%), *n*-hexadecanoic acid (6.93%), hexadecanoic acid, methyl ester (6.40%), benzenepropanoic acid, 3,5-bis(1,1-dimethylethyl)-4-hydroxy-, (5.21%), and phenol, 3,5-bis(1,1-dimethylethyl)- (5.10%). Among the 22 peaks observed in the GC-MS profile of *H. macroloba* methanol extract, only seven compounds were detected. The major compounds identified were *n*-hexadecanoic acid (16.16%), 3,7,11,15-tetramethyl-2-hexadecen-1-ol (12.76%), stigmast-5-en-3-ol, (3*β*,24S)-(6.73%), 1,2-benzenedicarboxylic acid, mono(2-ethylhexyl) ester (5.48%), benzenepropanoic acid, 3,5-*bis*(1,1-dimethylethyl)-4-hydroxy-, methyl ester (4.97%), and phenol, 2,4-bis(1,1-dimethylethyl) - (4.23%). The metabolites in *S. ilicifolium* were identified (Table 7) as 1,2-benzenedicarboxylic acid, mono(2-ethylhexyl) ester (7.69%), benzenepropanoic acid, 3,5-bis(1,1-dimethylethyl)-4-hydroxy-, methyl ester (7.03%), phenol, 3,5-bis(1,1-dimethylethyl)- (6.54%), and 3,7,11,15-tetramethyl-2-hexadecen-1-ol (6.24%), hexadecanoic acid, and methyl ester (5.28%).

## 4. Discussion

Photosynthetic pigments are important in the lives of plant species and are usually classified into three major categories: chlorophylls (*a*, *b*, *c*), carotenoids (carotene and xanthophylls), and phycobilins (phycoerythrin and phycocyanin) [22]. The major photosynthetic pigments studied in seaweed are presented as total carotenoid content and chlorophylls *a* and *b* expressed as *μ*g g^−1^ dry weight (DW). While chlorophylls comprise part of the components required for photosynthesis, the essential roles played by carotenoids are to pass light energy to chlorophyll and acting as strong antioxidants. The measurement in this study of higher carotenoids concentrations (ranging from 115.57 to 162.00 *μ*g g^−1^ DW) in the green seaweed (*H. macroloba* and *U. intestinalis*) is in concurrence with results reported by other researchers. This follows the suggestion that carotenoids are abundant in seaweed with higher photosynthetic activities such as green (Chlorophyta) and red (Rhodophyta) than in the brown (Phaeophyta) [23]. These results contrasted with a previous study where high carotenoid content was found in brown seaweed and low carotenoid content found in green seaweed [24]. The specific photosynthetic pigments and their concentrations are known to vary depending on their morphological structures and environmental factors [25]. However, useful information with regard to different habitats that may affect the biochemical composition of seaweed may be evident when a comparative study is conducted in closely related species. The relative abundance in this study of chlorophyll *a* content in 3 species investigated (varying between 141.98 and 313.09 *μ*gg^−1^ DW) agreed with those reported in other studies [26], in which the highest chlorophyll *a* content was measured in green seaweed *U. intestinalis* and the lowest measured in brown seaweed *S. ilicifolium*. As also noted in the present study, chlorophyll *b* was recorded to be the richest in green algae *U. intestinalis* and the poorest in brown algae *S. ilicifolium*.

The potential of extracts from marine seaweed for antidiabetic medication has attracted significant interest as a field of research [27]. The antidiabetic properties of seaweed through the inhibition of carbohydrate-hydrolysing enzymes have already been demonstrated in many studies, with *α*-glucosidase as one of the most effective methods for diabetes treatment [28,29].

Seaweed is known to comprise *a*-glucosidase inhibitors and one therapeutic approach for treating diabetes mellitus, and obesity is to retard the absorption of glucose via inhibition of *a*-glucosidase. Hwang et al. (2015) [30] found that the Taiwanese brown seaweed *S. hemiphyllum* had *a*-amylase and *a*-glucosidase (sucrose and maltase) inhibitory activity. Nagappan et al. 2017 [31] discovered that crude extract of *S. siliquosum* and *S. polycystum* collected from Port Dickson, Negeri Sembilan, Malaysia, inhibited *a*-glucosidase activity with IC_50_ value (0.57 ± 0.02 mg/ml for *S. siliquosum* and 0.69 ± 0.02 mg/mL for *S. polycystum*, respectively). Another researcher also reported the *α*-glucosidase inhibition of *S. ringgoldianum* with an IC_50_ value of 0.12 mg/mL and *Padina arborescens* (IC_50_ = 0.26 mg/mL) [32], while [33] Chin et al. 2015 found that water extracts of the green seaweed species *H. macroloba* showed inhibition activity against *a*-glucosidase with an IC_50_ value of 6.388 mg/mL.

The results of this study also demonstrated that morphological structures of seaweed species and the prevailing environmental factors (including sampling locations and times) [25] are important factors to consider for their antidiabetic properties and metabolites as emphasized by previous researchers.

Different solvent extracts used may possess different antidiabetic properties in the same species of seaweed [34]. Extracts in water may contain nonphenolic components and may have influenced glucosidase activity [35]. Besides, the contents and concentrations of bioactive compounds in the extracts of the seaweed studied may have been affected by the different polarities of the extracting solvents used [36]. The polyphenol content of crude extract of brown seaweed *Sargassum* sp. and *Ascophyllum nodosum* had been attributed to, for their high and mild *α*-amylase and *α*-glucosidase inhibitory activities [37, 38]. Polyphenols have the ability to chelate enzymes, causing them to precipitate and later experience alteration in structure coupled with a loss of their biological functions. Polyphenols from edible seaweed have also been suggested to influence responses relevant to diabetes through modulation of glucose-induced oxidative stress [39]. Therefore, phenolic groups which were reported in high concentrations in all the extracts in the present study could be correlated with the *α*-glucosidase inhibition found in the present study.

Fucoidan isolated from *Sargassum* sp. brown seaweed showed high *α*-glucosidase inhibitory activity [40]. Fucoidan delays the absorption of dietary carbohydrates in the intestine and leads to the suppression of blood glucose level after a meal. This may provide a way to regulate postprandial hyperglycaemia of diabetic patients. Although fucoidan was not measured in the present study, it is likely that in seaweed extracts which comprise complex matrixes of the compound, antidiabetic properties would not be closely connected with a specific compound, but with a mixture of polar and nonpolar compounds as this mixture of compounds acts synergistically. Nevertheless, our data revealed the potential of the extracts of *S. ilicifolium* and *H. macroloba* sp. to be used as efficient candidates for inhibiting the *α*-glucosidase enzyme after slight purification. According to Nguyen et al. 2011 [41], the search for *α*-glucosidase inhibitors from marine organisms is vital due to their ability to suppress the postprandial hyperglycaemia of diabetic patients.

The formation of cancer cells in the human body can be directly induced by free radicals. The use of natural anticancer drugs as effective chemopreventive agents has become an important approach in cancer prevention. Hence, radical scavenging compounds from marine algae can be used indirectly to reduce cancer formation in the human body [15]. A few researchers discovered the potential of green seaweed *Ulva* sp. as anticancer candidate. They reported the cytotoxicity activity of different *Ulva* sp. extracts towards different cell lines including human colon cancer (HCT 116) cells [42], human carcinoma of the nasopharynx cell line (KB), human hepatoma cell line (Bel-7402), and human lung adenocarcinoma epithelial cell line (A549) [43], and breast ductal carcinoma (T47D) cell line [44]. Other green seaweed species, *Halimeda tuna,* in chloroform and methanol extracts have also been reported to show cytotoxic activity against MCF-7 cells [45].

Furthermore, reports are few on the cytotoxicity of brown seaweed *Sargassum* sp. against cancer cell lines, including human liver hepatocellular carcinoma (HepG-2), human breast adenocarcinoma (MCF-7), human prostate cancer (LnCaP), breast ductal carcinoma (T47D) cell lines, and Jurkat cancer [46–50] with variations in the inhibition rates. The differences of cytotoxicity activity among different species of seaweed may be due to differences in species, chemical compositions, time and method of sampling, and extraction procedure [51].

Numerous studies have reported the role of carotenoids as potential anticancer candidates as carotenoids have been used as major phytonutrients for inhibiting the development of tumours *in vitro* and *in vivo*. This could be because carotenoids proved to play important roles such as preventing oxidative damage that is caused by free radicals or scavenging free radicals, inhibition of angiogenesis, prevention of cell propagation, apoptosis induction in lung, colon, breast, and prostate [52–56]. Several studies also reported that fucoxanthin (xanthophylls carotenoid) have inhibitory activities, antiproliferative effect, and ability to induce apoptosis in different cancer cell lines including T-cell leukaemia, leukaemia cells (HL-60) human colon cancer cells (Caco-2, HT-29, and DLD-1), and human prostate cancer cells (PC-3, DU 145, and LNCaP) [57–61]. Meanwhile, Ganesan et al. (2011) [62] also reported that siphonaxanthin (a marine carotenoid derived from green algae *Codium fragile*) is a growth-inhibitor against HL-60 cells.

Chlorophylls and their derivatives have been extensively studied with emphasis on their *in vitro* antimutagenic effects against numerous dietary and environmental mutagens [63] and were shown to possess anticancer properties and could play significant roles in cancer prevention [64, 65]. The concentrations of the contents of seaweed pigments (total carotenoids, chlorophylls *a* and *b*) in the present study clearly demonstrated that marine seaweed-derived pigments were important free radical scavengers able to diminish cancer development.

Active components responsible for various biological activities could be evaluated by investigating the chemical composition of each extract using GC-MS analysis. The highest number of compounds (27) was noted in the methanolic extract of *U. intestinalis,* followed by *H*. *macroloba* methanolic extract (22) and *S. ilicifolium* methanolic extract (24). Most of these compounds are known to exhibit various pharmacological activities. In the present study, the highest *α*-glucosidase inhibitory activity and cytotoxicity of the methanolic extract were related to the occurrence of a higher number of bioactive compounds including *n*-hexadecanoic acid, 3,7,11,15-tetramethyl-2-hexadecen-1-ol, stigmast-5-en-3-ol, (3*β*,24S)-, 1,2-benzenedicarboxylic acid, mono(2-ethylhexyl) ester, benzenepropanoic acid, 3,5-*bis*(1,1-dimethylethyl)-4-hydroxy-, methyl ester, and phenol, 2,4-bis(1,1-dimethylethyl)-.

Phytol (3,7,11,15-tetramethyl-2-hexadecen-1-ol) is an important diterpene that possesses antimicrobial, antioxidant, and anticancer activities [66, 67]. Pejin et al. [68] reported the cytotoxicity of phytol against seven tumour cells (MCF-7, PC-3 cells, HeLa, HT-29, A549, Hs294 T, and MDA-MB-231) and one normal cell of human origin. The workers found that MCF-7 and cervical HeLa cell lines were more sensitive to phytol compared to other cell lines. Phytol's ability for anticancer activity may be associated with its ability to remove the hydroxyl radical (free radical) [69].

The compound n-hexadecanoic acid is a common fatty acid that has been found in abundance in plants [70]. Previous studies reported that n-hexadecanoic acid selectively inhibits DNA topoisomerase-I and thus prevents the proliferation of human fibroblast cells [71]. Ravi and Krishnan [72] found that *Kigelia pinnata* leaves' crude extracts resulted in identification of n-hexadecanoic acid and showed significant cytotoxicity against human colorectal carcinoma cells (HCT-116). Nguyen et al. 2011 [41] reported that fatty acids with strong *a*-glucosidase-inhibitory activity, 7(Z)-octadecenoic acid and 7(Z) and 10(Z)-octadecadienoic acid, were purified and identified from sea cucumber. Another FA, 1,2-benzenedicarboxylic acid, mono(2-ethylhexyl) ester (BMEH) is known for antifungal, antidiabetic, anticancer, and antioxidant activities and as a potent antimicrobial agent [73, 74]. Selvakumar et al. 2019 [75] reported that BMEH isolated from marine *Streptomyces* sp. VITJS4 exhibited *in vitro* anticancer potential against liver (HepG2) cancer cells.

Stigmast-5-en-3-ol, (3*β*) which is also a form of fatty acid in plants was found in abundance in *H. macroloba*. Stigmast-5-en-3-ol, (3*β*) or *ß*-sterol is a phytosterol compound reported as the most common plant sterol, together with *ß*-sitosterol, and campesterol [76, 77] can act chemically as a compound with high antioxidant activity and a modest radical scavenger. Sujatha et al. 2010 [78] described the beneficial role of 3*β*-stigmast-5-en-3-ol in possessing antidiabetic property by stimulating glucose transport *in vitro*, in addition to its cholesterol-lowering efficacy. Benzenepropanoic acid, 3,5-bis(1,1-dimethylethyl)-4-hydroxy- methyl ester, on the other hand, exhibits antifungal and antioxidant activities [79].

## 5. Conclusion

*U. intestinalis, H. macroloba*, and *S. ilicifolium* seaweed species are potential sources of many bioactive, nutritionally and physiologically important compounds that could be utilized in nutraceuticals and pharmaceutical industries as sources of both food and medicine. The present study demonstrated that the contents of seaweed pigments (total carotenoids, chlorophylls *a* and *b*) have antidiabetic and anticancer potentials (and therefore, the biological activities resulting from their phytochemical content of bioactive compounds) and are not only based on species differences (whether brown, red, or green seaweed) but also depend, to a large extent, on the predominant environmental conditions at their sampling locations. Besides, further studies also need to be carried out to isolate, purify, and specifically investigate the efficacies and biological activities of all the identified compounds, and the current focus on the species to mine for new drugs is justified.

## Figures and Tables

**Table 1:** Sampling location for different species of seaweed.

Seaweed	Sampling locations
Sargassaceae	
*Sargassum ilicifolium* (Turner) C. Agardh (ID: IBS-SW1)	Teluk Kemang,
	Port Dickson
Halimedaceae	
*Halimeda macroloba* Decaisne (ID: IBS-SW2)	Merambung Shoal,
	Johore
Ulvaceae	
*Ulva intestinalis* (Linnaeus) Nees (ID: IBS-SW3)	Tanjung Adang, Johore

**Table 2:** The contents of carotenoids, chlorophyll a, and chlorophyll b from different species of seaweed.

Seaweed	Total carotenoid content (*μ*gg^−1^DW)	Chlorophyll *a* (*μ*gg^−1^ DW)	Chlorophyll *b* (*μ*gg^−1^ DW)
*Halimeda macroloba*	117.36 ± 1.30	154.42 ± 1.73	237.96 ± 8.25
*Ulva intestinalis*	162.00 ± 0.84	313.09 ± 2.53	292.52 ± 8.84
*Sargassum ilicifolium*	45.28 ± 1.77	141.98 ± 1.18	111.29 ± 2.28

Values are mean ± SEM, *n* = 3, dry weight (DW).

**Table 3:** The IC_50_ value of the *a*-glucosidase activities of the methanolic extract of *Halimeda macroloba*, *Ulva intestinalis*, *Sargassum ilicifolium*, and quercetin in ppm unit.

Samples	IC_50_ (ppm)
*Halimeda macroloba*	71.77 ± 2.33^*∗*^
*Ulva intestinalis*	>200
*Sargassum ilicifolium*	38.49 ± 0.58^*∗*^
Quercetin	16.53 ± 0.01

Results were presented as mean ± SEM. Values of ^*∗*^*P* < 0.05 were regarded as statistically significant.

**Table 4:** Potential cytotoxic activity of methalonic extracts of 3 seaweed species; *U. intestinalis, H. macroloba, and S. ilicifolium* on five different cell lines.

IC_50_ value (*μ*g/mL)					
	MCF-7 cells	MDA-MB 231	HT 29 cells	Hep G2 cells	3T3 cells
*Halimeda macroloba*	37.25 ± 0.58^*∗*^	33.24 ± 0.31^*∗*^	21.32 ± 0.25^*∗*^	47.40 ± 0.11^*∗*^	48.80 ± 0.11^*∗*^
*Ulva intestinalis*	46.10 ± 0.11^*∗*^	58.37 ± 0.47^*∗*^	42.230.11	23.21 ± 0.11^*∗*^	51.30 ± 0.14
*Sargassum ilicifolium*	44.00 ± 0.02	25.23 ± 0.11^*∗*^	37.03 ± 0.78^*∗*^	30.12 ± 0.91^*∗*^	52.13 ± 0.11^*∗*^

Each value is presented in means ± standard deviation (*n* = 6). Values within a row with different superscripts for each cancer cell line are significantly different (*P* < 0.05).

**Table 5:** Chemical composition of Ulva intestinalis.

No	Name	Retention time	Area, %	Molecular formula
*Phenol*				
1	Phenol, 3,5-bis(1,1-dimethylethyl)-	15.980	5.10	C_14_H_22_O
*Ketone*				
2	2-Pentadecanone, 6,10,14-trimethyl-	19.695	2.28	C_18_H_36_O
*Aldehyde*				
3	*E*-14-Hexadecenal	16.900	1.09	C_16_H_30_O
*Alcohol*				
4	3,7,11,15-Tetramethyl-2-hexadecen-1-ol	20.062	2.23	C_20_H_40_O
5	1-Dodecanol, 3,7,11-trimethyl-	22.334	1.17	C_15_H_32_O
*Hydrocarbons*				
6	8-Heptadecene	17.901	0.66	C_17_H_34_
7	1-Octadecene	19.160	0.79	C_18_H_36_
*Fatty acids*				
8	*n*-Hexadecanoic acid	20.981	6.93	C_16_H_32_O_2_
9	Oleic acid	22.664	3.26	C_18_H_34_O_2_
10	Octadecanoic acid	22.861	0.51	C_18_H_36_O_2_
11	15-Hydroxypentadecanoic acid	23.999	0.78	C_15_H_30_O_3_
	14-Pentadecenoic acid	26.733	1.51	C_15_H_28_O_2_
*Sterols*				
12	Stigmast-5-en-3-ol, oleate	30.028	1.24	C_47_H_82_O_2_
*Fatty acid methyl/ethyl esters*				
13	Cyclopentanetridecanoic acid, methyl ester	18.426	0.49	C_19_H_36_O_2_
14	Hexadecanoic acid, methyl ester	20.545	6.40	C_17_H_34_O_2_
15	9,12-Octadecadienoic acid, methyl ester	22.180	0.72	C_19_H_34_O_2_
16	9-Octadecenoic acid, methyl ester, (*E*)-	22.239	3.26	C_19_H_36_O_2_
17	11-Octadecenoic acid, methyl ester	22.297	0.81	C_19_H_36_O_2_
18	9,12-Octadecadienoic acid, methyl ester	22.180	0.72	C_19_H_34_O_2_
19	9-Octadecenoic acid, methyl ester, (*E*)-	22.239	3.26	C_19_H_36_O_2_
20	Octadecanoic acid, methyl ester	22.473	0.64	C_19_H_38_O_2_
21	Hexadecanoic acid, propyl ester	25.320	1.04	C_19_H_38_O_2_
*Other*				
22	Hexadecanoic acid, 2-hydroxy-1- (hydroxymethyl)ethyl ester	23.601	1.91	C_19_H_38_O_4_
23	Benzenepropanoic acid, 3,5-bis(1,1-dimethylethyl)-4-hydroxy-, methyl ester	20.642	5.21	C_18_H_28_O_3_
24	9-Octadecenamide, (*Z*)-	23.049	0.54	C_18_H_35_NO
25	Octadecanoic acid, 2,3-dihydroxypropyl ester	25.310	0.97	C_21_H_42_O_4_
26	Cyclohexanecarboxylic acid, undec-10-enyl ester	25.485	1.39	C_18_H_32_O_2_
27	1,2-Benzenedicarboxylic acid, mono(2-ethylhexyl) ester	25.946	7.61	C_16_H_22_O_4_

**Table 6:** Chemical composition of *Halimeda macroloba*.

No	Name	Retention time	Area, %	Molecular formula
*Phenol*				
1	Phenol, 2,4-bis(1,1-dimethylethyl)-	15.984	4.23	C_14_H_22_O
*Ketone*				
2	2-Pentadecanone, 6,10,14-trimethyl-	19.699	0.85	C_18_H_36_O
*Aldehyde*				
3	2,6-Octadienal, 3,7-dimethyl-, (*E*)-	12.760	0.45	C_10_H_16_O
*Alcohol*				
4	3,7,11,15-Tetramethyl-2-hexadecen-1-ol	19.625	12.76	C_20_H_40_O
*Hydrocarbons*				
5	3-Tetradecene, (*Z*)-	14.387	0.19	C_14_H_28_
6	1-Octadecene	19.160	0.79	C_18_H_36_
7	1-Tridecene	16.905	0.75	C_13_H_26_
8	Heptadecane	18.138	0.83	C_17_H_36_
*Fatty acids*				
9	Tetradecanoic acid	18.906	0.63	C_14_H_28_O_2_
10	*n*-Hexadecanoic acid	21.039	16.16	C_16_H_32_O_2_
11	9,12-Octadecadienoic acid (*Z, Z*)-	22.637	0.53	C_18_H_32_O_2_
12	Oleic acid	22.681	1.69	C_18_H_34_O_2_
13	15-Hydroxypentadecanoic acid	24.004	0.38	C_15_H_30_O_3_
*Sterols*				
14	Stigmasta-5,22-dien-3-ol, acetate, (3*β*)-	29.894	1.02	C_31_H_50_O_2_
15	Stigmast-5-en-3-ol, oleate	30.034	1.25	C_47_H_82_O_2_
16	Stigmast-5-en-3-ol, (3*β*,24S)-	31.487	6.73	C_29_H_50_O
17	Stigmast-4-en-3-one	32.366	0.84	C_29_H_48_O
*Fatty acid methyl/ethyl esters*				
18	Hexadecanoic acid, methyl ester	20.548	1.89	C_17_H_34_O_2_
19	9,12-Octadecadienoic acid, methyl ester	22.185	0.44	C_19_H_34_O_2_
20	9-Octadecenoic acid (*Z*)-, methyl ester	22.242	1.55	C_19_H_36_O_2_
*Other*				
21	Benzenepropanoic acid, 3,5-*bis*(1,1-dimethylethyl)-4-hydroxy-, methyl ester	20.647	4.97	C_18_H_28_O_3_
22	1,2-Benzenedicarboxylic acid, mono(2-ethylhexyl) ester	25.949	5.48	C_16_H_22_O_4_

**Table 7:** Chemical composition of *Sargassum ilicifolium*.

No	Name	Retention time	Area, %	Molecular formula
*Phenol*				
1	Phenol, 3,5-bis(1,1-dimethylethyl)-	15.982	6.54	C_14_H_22_O
*Ketone*				
2	2-Pentadecanone, 6,10,14-trimethyl-	19.696	0.85	C_18_H_36_O
*Aldehyde*				
3	*E*-14-Hexadecenal	16.903	1.65	C_16_H_30_O
4	Octadecanal	17.937	0.43	C_18_H_36_O
*Alcohol*				
5	3,7,11,15-Tetramethyl-2-hexadecen-1-ol	20.067	6.24	C_20_H_40_O
6	1-Dodecanol, 3,7,11-trimethyl-	22.337	2.84	C_15_H_32_O
*Hydrocarbons*				
7	1-Tetradecene	14.385	0.52	C_14_H_28_
8	1-Octadecene	19.161	0.83	C_18_H_36_
9	1-Nonadecene	21.206	0.30	C_19_H_38_
*Fatty acids*				
10	*n*-Hexadecanoic acid	20.963	1.20	C_16_H_32_O_2_
*Sterols*				
11	Stigmasta-5,22-dien-3-ol, acetate, (3*β*)-	30.024	0.83	C_31_H_50_O_2_
12	Cholest-4-en-3-one	31.086	0.87	C_27_H_44_O
13	Stigmasta-4,24(28)-dien-3-one, (24*E*)-	32.357	1.52	C_29_H_46_O
*Fatty acid methyl/ethyl esters*				
14	Tridecanoic acid, 12-methyl-, methyl ester	18.427	0.53	C_15_H_30_O_2_
15	Hexadecanoic acid, methyl ester	20.546	5.28	C_17_H_34_O_2_
16	9,12-Octadecadienoic acid, methyl ester	22.181	0.76	C_19_H_34_O_2_
17	9-Octadecenoic acid, methyl ester, (*E*)-	22.240	3.70	C_19_H_36_O_2_
18	11-Octadecenoic acid, methyl ester, (*Z*)-	22.290	0.77	C_19_H_36_O_2_
19	Octadecanoic acid, methyl ester	22.473	0.54	C_19_H_38_O_2_
20	Hexadecanoic acid, 2,3-dihydroxypropyl ester	25.319	0.66	C_19_H_38_O_4_
*Others*				
21	Benzenepropanoic acid, 3,5-bis(1,1-dimethylethyl)-4-hydroxy-, methyl ester	20.645	7.03	C_18_H_28_O_3_
22	Hexadecanoic acid, 2-hydroxy-1- (hydroxymethyl)ethyl ester	23 .601	1.17	C_19_H_38_O_4_
23	1,2-Benzenedicarboxylic acid, mono(2-ethylhexyl) ester	25.946	7.69	C_16_H_22_O_4_
24	Squalene	27.992	0.74	C_30_H_50_

## Data Availability

All data that support the findings of this study are included in this published article.
